# A second polymorph of (2*E*)-1-(4-fluoro­phen­yl)-3-(3,4,5-trimethoxy­phen­yl)prop-2-en-1-one

**DOI:** 10.1107/S1600536809028517

**Published:** 2009-07-25

**Authors:** Jerry P. Jasinski, Ray J. Butcher, K. Veena, B. Narayana, H. S. Yathirajan

**Affiliations:** a Department of Chemistry, Keene State College, 229 Main Street, Keene, NH 03435-2001, USA; b Department of Chemistry, Howard University, 525 College Street NW, Washington DC 20059, USA; c Department of Studies in Chemistry, Mangalore University, Mangalagangotri 574 199, India; d Department of Studies in Chemistry, University of Mysore, Manasagangotri, Mysore 570 006, India

## Abstract

The crystal structure of the title compound, C_18_H_17_FO_4_, reported here is a polymorph of the structure first reported by Patil *et al.* [*Mol. Cryst. Liq. Cryst. Sci. Technol. Sect. A* (2007), **461**, 123–130]. It is a chalcone analog and consists of substituted phenyl rings bonded at the opposite ends of a propenone group, the biologically active region. The dihedral angle between the mean planes of the aromatic rings within the 4-fluoro­phenyl and trimethoxy­phenyl groups is 28.7 (1)° compared to 20.8 (6)° in the published structure. The angles between the mean plane of the prop-2-ene-1-one group and the mean plane of aromatic rings within the 4-fluoro­phenyl and trimethoxy­phenyl groups are 30.3 (4) and 7.4 (7)°, respectively, in contast to 10.7 (3) and 12.36° for the polymorph. While the two 3-meth­oxy groups are in the plane of the trimeth­oxy-substituted ring, the 4-meth­oxy group is in a synclinical [−*sc* = −78.1 (2)°] or anti­clinical [+*ac* = 104.0 (4)°] position, compared to a +*sc* [53.0 (4)°] or −*ac* [−132.4 (7)°] position. While no classical hydrogen bonds are present, weak inter­molecular C—H⋯π-ring inter­actions are observed which contribute to the stability of the crystal packing. The two polymorphs crystallize in the same space group, *P*2_1_/*c*, but have different cell parameters for the *a*, *b* and *c* axes and the β angle. A comparison of the mol­ecular geometries of both polymorphs to a geometry optimized density functional theory (DFT) calculation at the B3-LYP/6–311+G(d,p) level for each structure provides additional support to these observations.

## Related literature

For general background to the biological activity of similar compounds, see: Dimmock *et al.* (1999 ▶); Lin *et al.* (2002 ▶); Nakamura *et al.* (2002 ▶); Nowakowska (2007 ▶); Opletalova & Sedivy (1999 ▶). For related structures, see: Butcher *et al.* (2006 ▶, 2007 ▶); Chopra *et al.* (2007 ▶); Fun *et al.* (2008 ▶); Jasinski *et al.* (2009 ▶); Patil *et al.* (2007 ▶); Qiu *et al.* (2006 ▶); Teh *et al.* (2007 ▶). For density functional theory (DFT), see: Becke (1988 ▶, 1993 ▶); Hehre *et al.* (1986 ▶); Lee *et al.* (1988 ▶); Schmidt & Polik (2007 ▶). For a description of the Cambridge Structural Database, see: Allen (2002 ▶). For the *GAUSSIAN03* program package, see: Frisch *et al.* (2004 ▶).
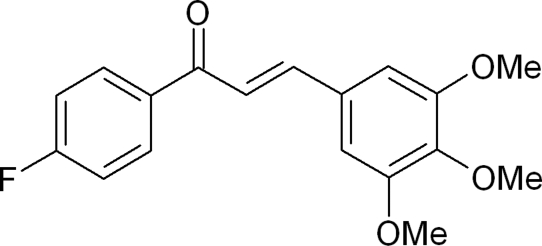


## Experimental

### 

#### Crystal data

C_18_H_17_FO_4_
*M* *_r_* = 316.32
Monoclinic, 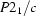

*a* = 12.4250 (2) Å
*b* = 8.6280 (1) Å
*c* = 14.9038 (2) Å
β = 98.3217 (12)°
*V* = 1580.91 (4) Å^3^
*Z* = 4
Cu *K*α radiation
μ = 0.85 mm^−1^
*T* = 295 K
0.47 × 0.40 × 0.22 mm

#### Data collection

Oxford Diffraction Gemini R diffractometer
Absorption correction: multi-scan (*CrysAlis RED*; Oxford Diffraction, 2007 ▶) *T*_min_ = 0.557, *T*_max_ = 0.830
8137 measured reflections
3216 independent reflections
2396 reflections with *I* > 2σ(*I*)
*R*_int_ = 0.018

#### Refinement

*R*[*F* ^2^ > 2σ(*F* ^2^)] = 0.040
*wR*(*F* ^2^) = 0.126
*S* = 1.10
3216 reflections
211 parameters
H-atom parameters constrained
Δρ_max_ = 0.13 e Å^−3^
Δρ_min_ = −0.18 e Å^−3^

### 

Data collection: *CrysAlis Pro* (Oxford Diffraction, 2007 ▶); cell refinement: *CrysAlis RED* (Oxford Diffraction, 2007 ▶); data reduction: *CrysAlis RED*; program(s) used to solve structure: *SHELXS97* (Sheldrick, 2008 ▶); program(s) used to refine structure: *SHELXL97* (Sheldrick, 2008 ▶); molecular graphics: *SHELXTL* (Sheldrick, 2008 ▶); software used to prepare material for publication: *SHELXTL*.

## Supplementary Material

Crystal structure: contains datablocks global, I. DOI: 10.1107/S1600536809028517/sj2632sup1.cif

Structure factors: contains datablocks I. DOI: 10.1107/S1600536809028517/sj2632Isup2.hkl

Additional supplementary materials:  crystallographic information; 3D view; checkCIF report

## Figures and Tables

**Table 1:** Hydrogen-bond geometry (Å, °)

*D*—H⋯*A*	*D*—H	H⋯*A*	*D*⋯*A*	*D*—H⋯*A*
C3—H3*A*⋯*Cg*2^i^	0.93	2.91	3.6571 (19)	138

Symmetry code: (i) 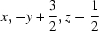. *Cg*2 is the centroid of the C10–C15 ring.

**Fig. 1.**
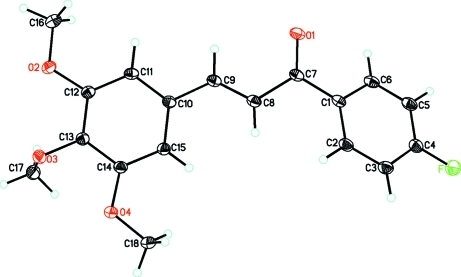
Molecular structure of C18H17FO4 showing the atom labeling scheme and 50% probability displacement ellipsoids.

**Fig. 2.**
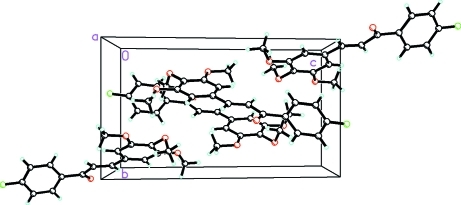
Packing diagram of the title compound, (I), viewed down the a axis.

**Fig. 3.**
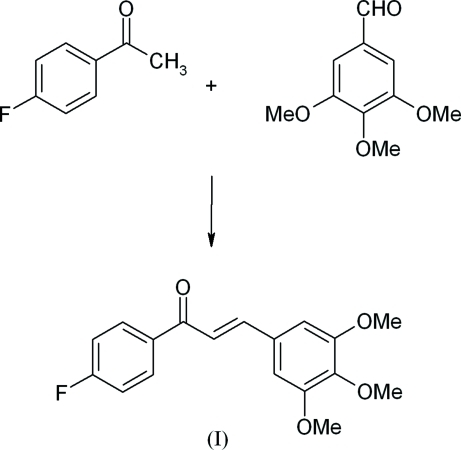
The formation of the title compound.

## supplementary crystallographic information

### Comment

Chalcones are unique molecules with significant biological activity (Dimmock *et al.* 1999). Chalcones and their analogs have been shown to have potential antifungal (Opletalova & Sedivy, 1999), anti-tuberculosis (Lin *et al.* 2002), anti-infective and anti-inflammatory properties (Nowakowska, 2007). The synthesis and biological activity of some fluorinated chalcone derivatives have also been reported (Nakamura *et al.* 2002). Structures of a series of substituted (2E)-3-(2-fluoro-4-phenoxyphenyl)-1-phenylprop-2-en-1-ones have also been reported. (Chopra *et al.* 2007).  As a continuation of our work on chalcones (Jasinski *et al.* 2009) and in view of the importance of fluoro-chalcones, this paper describes a new polymorphic form of (I), C_18_H_17_FO_4_, (2E)-1-(4-fluorophenyl)-3-(3,4,5-trimethoxyphenyl)prop-2-en-1-one, first reported by Patil *et al.* (2007).  Substantial changes in the cell parameters provides solid support for the recognition of this new polymorphic form for (I).

The title compound,(I), is a chalcone analog and consists of substituted phenyl rings bonded at the opposite ends of a propenone moiety, the biologically active region (Fig. 1).  The dihedral angle between the mean planes of the phenyl rings with the 4-fluorophenyl and trimethoxyphenyl substituents is 28.7 (1)° compared to 20.8 (6)° in the  polymorph.  The angles between the mean plane of the prop-2-ene-1-one group and those of the 4-fluorophenyl and trimethoxyphenyl rings are 30.3 (4)° and 7.4 (7)°, respectively, compared to 10.7 (3)° and 12.36° as reported by Patil *et al* (2007).  While the two *meta* -methoxy groups are in the plane of the trimethoxy substituted phenyl ring, the *para* -methoxy group is in a synclinical (-sc) (torsion angle C(12)-C(13)-C(17)-O(3) = -78.1 (2)°) or anticlinical (+ac) (torsion angle C(14)-C(13)-C(17)-O(3) = 104.0 (4)°) orientation, compared to the (+sc) (torsion angle C(12)-C(13)-C(17)-O(3) = 53.0 (4)°) or -ac (torsion angle C(14)-C(13)-C(17)-O(3) =-132.4 (7)°) orientation as reported by Patil *et al.* (2007).  While no classical hydrogen bonds are present, weak C(3)-H(3A)···Cg2 [C(3)-H(3A)···Cg2 = 138°; C(3)···Cg2 = 3.6571 (19) Å; x,3/2-y, -1/2+z; where Cg2 = C(10)-C(15)] C—H···π-ring intermolecular interactions are observed which contribute to the stability of the crystal packing (Fig. 2).  The two polymorphs crystallize in the same space group,*P2_1_/c*, but have different cell parameters for the *a* [12.4250 (2)Å vs 7.693 (0)Å], *b* [8.62800 (10)Å vs 15.232 (1)Å], *c* [14.9038 (2)Å vs 14.128 (1)Å] axes and β angle [98.3217 (12)° vs 106.60 (0)°].

A geometry optimized density functional theory (DFT) calculation (Schmidt & Polik, 2007) was performed for each of the two polymorphs, with the *GAUSSIAN03* program package (Frisch *et al.* 2004) employing the B3-LYP (Becke three parameter Lee-Yang-Parr) exchange correlation functional, which combines the hybrid exchange functional of Becke (Becke, 1988,1993) with the gradient-correlation functional of Lee, Yang and Parr (Lee *et al.* 1988) and the 6–311+G(d,p) basis set (Hehre *et al.* 1986). Starting geometries were taken from X-ray refinement data for (I) and from coordinates from the Cambridge Structural Database (CSD) (Allen, 2002) for the Patil *et al.* (2007)  structure (SIRDUT). Interestingly, both structures converged to nearly the same geometric state. The dihedral angle between the mean planes of the phenyl rings within the 4-fluorophenyl and trimethoxyphenyl groups became 18.0 (9)° compared to 19.3 (6)° (SIRDUT). The angle between the mean plane of the prop-2-ene-1-one group and the mean plane of phenyl rings within the 4-fluorophenyl and trimethoxyphenyl groups became 14.0 (3)° and 5.2 (3)°, respectively, *versus* 14.4 (9)° and 5.2 (5)° (SIRDUT), significantly different from that observed in the crystalline state for each polymorph. In addition, the *para* methoxy group became synclinical (-*sc*) (torsion angle C(12)—C(13)—C(17)—O(3) = -77.8 (2)°) or anticlinical (+*ac*) (torsion angle C(14)—C(13)—C(17)—O(3) = 106.2 (8)°) in (I), compared to a (+*sc*) (torsion angle C(12)—C(13)—C(17)—O(3) = 79.2 (4)°°) or -*ac* (torsion angle C(14)—C(13)—C(17)—O(3) = -104.9 (5)°) in SIRDUT. It is clear that each polymeric form adjusted itself in different ways to achieve the DFT calculated geometric state. Bond distances and bond angles are relatively unchanged between the DFT calculated values and the observed values in (I) and SIRDUT with the exception of the *para* methoxy group as described earlier.

### Experimental

The title compound was synthesized by the reported procedure (Patil *et al*., 2007). The solid product obtained was filtered and recrystallized from ethanol. X-ray quality crystals were grown from ethyl acetate solution by slow evaporation (m.p.: 362-364 K). Analysis for C_18_H_17_FO_4_: Found (calculated): C: 68.27 (68.35%); H:5.36 (5.42%).

### Refinement

All of the H atoms were placed in their calculated positions and then refined using the riding model with C—H = 0.93–0.96 Å, and with *U*_iso_(H) = 1.18–1.50 *U*_eq_(C).

### Crystal data

**Table:**

C_18_H_17_FO_4_	*F*(000) = 664
*M**_r_* = 316.32	*D*_x_ = 1.329 Mg m^−^^3^
Monoclinic, *P*2_1_/*c*	Cu *K*α radiation, λ = 1.54184 Å
Hall symbol: -P 2ybc	Cell parameters from 4493 reflections
*a* = 12.4250 (2) Å	θ = 4.3–77.3°
*b* = 8.6280 (1) Å	µ = 0.85 mm^−^^1^
*c* = 14.9038 (2) Å	*T* = 295 K
β = 98.3217 (12)°	Prism, colorless
*V* = 1580.91 (4) Å^3^	0.47 × 0.40 × 0.22 mm
*Z* = 4	

### Data collection

**Table:**

Oxford Diffraction Gemini R diffractometer	3216 independent reflections
Radiation source: fine-focus sealed tube	2396 reflections with *I* > 2σ(*I*)
graphite	*R*_int_ = 0.018
Detector resolution: 10.5081 pixels mm^-1^	θ_max_ = 77.9°, θ_min_ = 5.9°
φ and ω scans	*h* = −14→15
Absorption correction: multi-scan (*CrysAlis RED*; Oxford Diffraction, 2007)	*k* = −10→9
*T*_min_ = 0.557, *T*_max_ = 0.830	*l* = −18→18
8137 measured reflections	

### Refinement

**Table:**

Refinement on *F*^2^	Primary atom site location: structure-invariant direct methods
Least-squares matrix: full	Secondary atom site location: difference Fourier map
*R*[*F*^2^ > 2σ(*F*^2^)] = 0.040	Hydrogen site location: inferred from neighbouring sites
*wR*(*F*^2^) = 0.126	H-atom parameters constrained
*S* = 1.10	*w* = 1/[σ^2^(*F*_o_^2^) + (0.0683*P*)^2^ + 0.1035*P*] where *P* = (*F*_o_^2^ + 2*F*_c_^2^)/3
3216 reflections	(Δ/σ)_max_ < 0.001
211 parameters	Δρ_max_ = 0.13 e Å^−^^3^
0 restraints	Δρ_min_ = −0.18 e Å^−^^3^

### Special details

**Table:**

Geometry. All e.s.d.'s (except the e.s.d. in the dihedral angle between two l.s. planes) are estimated using the full covariance matrix. The cell e.s.d.'s are taken into account individually in the estimation of e.s.d.'s in distances, angles and torsion angles; correlations between e.s.d.'s in cell parameters are only used when they are defined by crystal symmetry. An approximate (isotropic) treatment of cell e.s.d.'s is used for estimating e.s.d.'s involving l.s. planes.
Refinement. Refinement of *F*^2^ against ALL reflections. The weighted *R*-factor *wR* and goodness of fit *S* are based on *F*^2^, conventional *R*-factors *R* are based on *F*, with *F* set to zero for negative *F*^2^. The threshold expression of *F*^2^ > σ(*F*^2^) is used only for calculating *R*-factors(gt) *etc*. and is not relevant to the choice of reflections for refinement. *R*-factors based on *F*^2^ are statistically about twice as large as those based on *F*, and *R*- factors based on ALL data will be even larger.

### Fractional atomic coordinates and isotropic or equivalent isotropic displacement parameters (Å^2^)

**Table:**

	*x*	*y*	*z*	*U*_iso_*/*U*_eq_	
O4	0.54332 (9)	0.78663 (17)	0.56646 (8)	0.0813 (4)	
F	0.12194 (11)	0.38071 (16)	−0.04098 (8)	0.0982 (4)	
O1	0.01131 (9)	0.41222 (15)	0.35678 (8)	0.0737 (3)	
O2	0.33800 (9)	0.64264 (16)	0.79007 (7)	0.0751 (3)	
O3	0.51516 (9)	0.76879 (15)	0.74012 (8)	0.0716 (3)	
C1	0.10184 (11)	0.43862 (16)	0.22896 (11)	0.0576 (3)	
C2	0.16144 (13)	0.54012 (19)	0.18363 (12)	0.0684 (4)	
H2A	0.1980	0.6219	0.2152	0.082*	
C3	0.16769 (15)	0.5225 (2)	0.09261 (12)	0.0745 (4)	
H3A	0.2071	0.5920	0.0625	0.089*	
C4	0.11452 (13)	0.4002 (2)	0.04776 (12)	0.0698 (4)	
C5	0.05441 (14)	0.2970 (2)	0.08954 (14)	0.0765 (5)	
H5A	0.0189	0.2149	0.0574	0.092*	
C6	0.04768 (13)	0.31760 (19)	0.17996 (13)	0.0695 (4)	
H6A	0.0061	0.2493	0.2090	0.083*	
C7	0.09279 (11)	0.45582 (16)	0.32705 (11)	0.0591 (3)	
C8	0.18590 (12)	0.52523 (19)	0.38636 (11)	0.0633 (4)	
H8A	0.2441	0.5643	0.3605	0.076*	
C9	0.18866 (11)	0.53327 (18)	0.47527 (11)	0.0612 (4)	
H9A	0.1275	0.4964	0.4978	0.073*	
C10	0.27643 (11)	0.59319 (17)	0.54241 (10)	0.0572 (3)	
C11	0.26418 (11)	0.58419 (18)	0.63367 (10)	0.0605 (4)	
H11A	0.2019	0.5398	0.6505	0.073*	
C12	0.34453 (11)	0.64123 (18)	0.69953 (10)	0.0587 (3)	
C13	0.43756 (12)	0.70811 (18)	0.67455 (10)	0.0591 (3)	
C14	0.44971 (11)	0.71778 (19)	0.58304 (10)	0.0611 (4)	
C15	0.36992 (12)	0.66065 (19)	0.51690 (10)	0.0611 (4)	
H15A	0.3783	0.6670	0.4560	0.073*	
C16	0.25626 (17)	0.5508 (3)	0.82150 (13)	0.0855 (5)	
H16A	0.1858	0.5875	0.7950	0.128*	
H16B	0.2630	0.5581	0.8863	0.128*	
H16C	0.2647	0.4448	0.8044	0.128*	
C17	0.60831 (14)	0.6733 (3)	0.75995 (13)	0.0836 (5)	
H17A	0.5874	0.5746	0.7817	0.125*	
H17B	0.6597	0.7219	0.8056	0.125*	
H17C	0.6410	0.6586	0.7060	0.125*	
C18	0.56292 (15)	0.7943 (3)	0.47481 (13)	0.0864 (6)	
H18A	0.5608	0.6918	0.4496	0.130*	
H18B	0.6332	0.8392	0.4727	0.130*	
H18C	0.5080	0.8571	0.4403	0.130*	

### Atomic displacement parameters (Å^2^)

**Table:**

	*U*^11^	*U*^22^	*U*^33^	*U*^12^	*U*^13^	*U*^23^
O4	0.0623 (7)	0.1152 (10)	0.0677 (7)	−0.0271 (7)	0.0134 (5)	−0.0012 (6)
F	0.1049 (8)	0.1062 (9)	0.0868 (7)	−0.0161 (7)	0.0246 (6)	−0.0256 (6)
O1	0.0523 (6)	0.0813 (8)	0.0886 (8)	−0.0117 (5)	0.0137 (5)	0.0053 (6)
O2	0.0684 (7)	0.0970 (8)	0.0630 (6)	−0.0017 (6)	0.0201 (5)	0.0015 (6)
O3	0.0601 (6)	0.0861 (8)	0.0679 (6)	−0.0012 (5)	0.0075 (5)	−0.0097 (5)
C1	0.0414 (6)	0.0491 (7)	0.0814 (9)	−0.0007 (6)	0.0063 (6)	−0.0042 (6)
C2	0.0646 (9)	0.0594 (9)	0.0810 (10)	−0.0177 (7)	0.0099 (7)	−0.0098 (7)
C3	0.0732 (10)	0.0666 (10)	0.0854 (11)	−0.0157 (8)	0.0173 (8)	−0.0035 (8)
C4	0.0600 (8)	0.0715 (10)	0.0785 (10)	−0.0014 (7)	0.0121 (7)	−0.0144 (8)
C5	0.0625 (9)	0.0657 (10)	0.1020 (13)	−0.0142 (8)	0.0148 (8)	−0.0260 (9)
C6	0.0559 (8)	0.0569 (8)	0.0981 (12)	−0.0121 (7)	0.0188 (8)	−0.0114 (8)
C7	0.0456 (7)	0.0498 (7)	0.0817 (9)	0.0004 (6)	0.0092 (6)	0.0014 (6)
C8	0.0479 (7)	0.0645 (9)	0.0787 (10)	−0.0036 (6)	0.0135 (6)	−0.0061 (7)
C9	0.0474 (7)	0.0602 (8)	0.0763 (9)	0.0007 (6)	0.0098 (6)	0.0070 (7)
C10	0.0474 (7)	0.0563 (8)	0.0683 (8)	0.0046 (6)	0.0094 (6)	0.0031 (6)
C11	0.0493 (7)	0.0617 (8)	0.0728 (9)	0.0039 (6)	0.0167 (6)	0.0076 (7)
C12	0.0522 (7)	0.0622 (8)	0.0633 (8)	0.0106 (6)	0.0137 (6)	0.0038 (6)
C13	0.0511 (7)	0.0624 (8)	0.0645 (8)	0.0053 (6)	0.0103 (6)	−0.0023 (6)
C14	0.0486 (7)	0.0690 (9)	0.0670 (9)	−0.0016 (6)	0.0128 (6)	0.0014 (7)
C15	0.0527 (7)	0.0716 (9)	0.0600 (8)	0.0002 (7)	0.0113 (6)	0.0022 (7)
C16	0.0891 (12)	0.0958 (13)	0.0777 (11)	−0.0033 (10)	0.0325 (9)	0.0095 (9)
C17	0.0593 (9)	0.1147 (15)	0.0747 (11)	0.0075 (10)	0.0026 (8)	−0.0003 (10)
C18	0.0681 (10)	0.1196 (16)	0.0749 (11)	−0.0239 (11)	0.0224 (8)	0.0043 (10)

### Geometric parameters (Å, °)

**Table:**

O4—C14	1.3602 (18)	C8—H8A	0.9300
O4—C18	1.423 (2)	C9—C10	1.463 (2)
F—C4	1.350 (2)	C9—H9A	0.9300
O1—C7	1.2218 (18)	C10—C11	1.393 (2)
O2—C12	1.3635 (18)	C10—C15	1.400 (2)
O2—C16	1.420 (2)	C11—C12	1.385 (2)
O3—C13	1.3734 (19)	C11—H11A	0.9300
O3—C17	1.417 (2)	C12—C13	1.390 (2)
C1—C2	1.384 (2)	C13—C14	1.396 (2)
C1—C6	1.391 (2)	C14—C15	1.384 (2)
C1—C7	1.490 (2)	C15—H15A	0.9300
C2—C3	1.378 (2)	C16—H16A	0.9600
C2—H2A	0.9300	C16—H16B	0.9600
C3—C4	1.367 (2)	C16—H16C	0.9600
C3—H3A	0.9300	C17—H17A	0.9600
C4—C5	1.368 (3)	C17—H17B	0.9600
C5—C6	1.374 (3)	C17—H17C	0.9600
C5—H5A	0.9300	C18—H18A	0.9600
C6—H6A	0.9300	C18—H18B	0.9600
C7—C8	1.477 (2)	C18—H18C	0.9600
C8—C9	1.322 (2)		
			
C14—O4—C18	117.66 (13)	C12—C11—C10	120.19 (13)
C12—O2—C16	117.99 (14)	C12—C11—H11A	119.9
C13—O3—C17	113.25 (13)	C10—C11—H11A	119.9
C2—C1—C6	118.10 (15)	O2—C12—C11	124.36 (13)
C2—C1—C7	122.51 (13)	O2—C12—C13	115.61 (13)
C6—C1—C7	119.38 (13)	C11—C12—C13	119.98 (13)
C3—C2—C1	121.37 (15)	O3—C13—C12	119.54 (13)
C3—C2—H2A	119.3	O3—C13—C14	120.55 (13)
C1—C2—H2A	119.3	C12—C13—C14	119.88 (14)
C4—C3—C2	118.33 (16)	O4—C14—C15	124.71 (14)
C4—C3—H3A	120.8	O4—C14—C13	114.82 (13)
C2—C3—H3A	120.8	C15—C14—C13	120.47 (13)
F—C4—C3	118.60 (16)	C14—C15—C10	119.45 (14)
F—C4—C5	118.96 (15)	C14—C15—H15A	120.3
C3—C4—C5	122.45 (16)	C10—C15—H15A	120.3
C4—C5—C6	118.50 (15)	O2—C16—H16A	109.5
C4—C5—H5A	120.8	O2—C16—H16B	109.5
C6—C5—H5A	120.8	H16A—C16—H16B	109.5
C5—C6—C1	121.24 (15)	O2—C16—H16C	109.5
C5—C6—H6A	119.4	H16A—C16—H16C	109.5
C1—C6—H6A	119.4	H16B—C16—H16C	109.5
O1—C7—C8	121.74 (15)	O3—C17—H17A	109.5
O1—C7—C1	120.62 (13)	O3—C17—H17B	109.5
C8—C7—C1	117.64 (12)	H17A—C17—H17B	109.5
C9—C8—C7	121.69 (14)	O3—C17—H17C	109.5
C9—C8—H8A	119.2	H17A—C17—H17C	109.5
C7—C8—H8A	119.2	H17B—C17—H17C	109.5
C8—C9—C10	127.76 (14)	O4—C18—H18A	109.5
C8—C9—H9A	116.1	O4—C18—H18B	109.5
C10—C9—H9A	116.1	H18A—C18—H18B	109.5
C11—C10—C15	120.04 (13)	O4—C18—H18C	109.5
C11—C10—C9	118.17 (13)	H18A—C18—H18C	109.5
C15—C10—C9	121.77 (13)	H18B—C18—H18C	109.5
			
C6—C1—C2—C3	−0.2 (2)	C16—O2—C12—C11	14.4 (2)
C7—C1—C2—C3	−179.40 (14)	C16—O2—C12—C13	−168.15 (15)
C1—C2—C3—C4	−0.9 (3)	C10—C11—C12—O2	177.51 (14)
C2—C3—C4—F	−178.84 (16)	C10—C11—C12—C13	0.2 (2)
C2—C3—C4—C5	1.0 (3)	C17—O3—C13—C12	104.04 (17)
F—C4—C5—C6	179.78 (15)	C17—O3—C13—C14	−78.13 (19)
C3—C4—C5—C6	−0.1 (3)	O2—C12—C13—O3	0.4 (2)
C4—C5—C6—C1	−1.0 (3)	C11—C12—C13—O3	177.92 (13)
C2—C1—C6—C5	1.2 (2)	O2—C12—C13—C14	−177.47 (13)
C7—C1—C6—C5	−179.61 (15)	C11—C12—C13—C14	0.1 (2)
C2—C1—C7—O1	149.90 (16)	C18—O4—C14—C15	−2.9 (3)
C6—C1—C7—O1	−29.3 (2)	C18—O4—C14—C13	177.40 (16)
C2—C1—C7—C8	−30.8 (2)	O3—C13—C14—O4	1.6 (2)
C6—C1—C7—C8	150.00 (14)	C12—C13—C14—O4	179.41 (14)
O1—C7—C8—C9	4.9 (2)	O3—C13—C14—C15	−178.09 (15)
C1—C7—C8—C9	−174.35 (14)	C12—C13—C14—C15	−0.3 (2)
C7—C8—C9—C10	177.60 (14)	O4—C14—C15—C10	−179.47 (15)
C8—C9—C10—C11	−177.15 (15)	C13—C14—C15—C10	0.2 (2)
C8—C9—C10—C15	4.0 (2)	C11—C10—C15—C14	0.1 (2)
C15—C10—C11—C12	−0.3 (2)	C9—C10—C15—C14	178.96 (14)
C9—C10—C11—C12	−179.18 (14)		

### Hydrogen-bond geometry (Å, °)

**Table:**

*D*—H···*A*	*D*—H	H···*A*	*D*···*A*	*D*—H···*A*
C3—H3A···Cg2^i^	0.93	2.91	3.6571 (19)	138

Symmetry codes: (i) *x*, −*y*+3/2, *z*−1/2.
